# Draft Genome Sequence of Streptococcus anginosus UMB0839, Isolated from the Female Urinary Tract

**DOI:** 10.1128/MRA.00411-20

**Published:** 2020-06-04

**Authors:** Thomas Scott, Taylor Miller-Ensminger, Adelina Voukadinova, Alan J. Wolfe, Catherine Putonti

**Affiliations:** a Bioinformatics Program, Loyola University Chicago, Chicago, Illinois, USA; b Department of Biology, Loyola University Chicago, Chicago, Illinois, USA; c Department of Microbiology and Immunology, Stritch School of Medicine, Loyola University Chicago, Maywood, Illinois, USA; d Department of Computer Science, Loyola University Chicago, Chicago, Illinois, USA; Portland State University

## Abstract

Here, we present the draft genome sequence of Streptococcus anginosus UMB0839, isolated from the female urinary tract. The total size of the genome is 2,104,786 bp assembled into 42 contigs with a GC content of 38.8% and 284× genome coverage.

## ANNOUNCEMENT

Streptococcus anginosus is a member of the microbiota of the oral cavity, throat, and urinary tract. Previous studies on *S. anginosus* and other members of the *S. milleri* group (which also includes S. intermedius and S. constellatus) reported an association with purulent infection in both adults and children (1, 2). Compared to other members of the *S. milleri* group, *S. anginosus* is most abundant in the urogenital tract and is associated with urinary tract infections (1–3) and urgency urinary incontinence (4). Here, we sequenced an *S. anginosus* strain (UMB0839) isolated from a catheterized urine sample obtained from a pregnant woman.

The Streptococcus anginosus strain was collected as part of a prior institutional review board (IRB)-approved study (5), following the expanded quantitative urinary culture (EQUC) protocol, as previously described (6). The genus and species of the isolate were determined through matrix-assisted laser desorption ionization–time of flight (MALDI-TOF) mass spectrometry (6), after which it was stored at −80°C. The *S. anginosus* isolate was taken from the freezer and streaked onto a Columbia nalidixic acid (CNA) agar plate and incubated at 35°C with 5% CO_2_ for 24 h. After incubation, a single colony was selected and inoculated in a liquid culture LB broth at 37°C with shaking for 24 h. The DNA was extracted using the Qiagen DNeasy blood and tissue kit, following the protocol for Gram-positive bacteria with slight modifications; 230 μl of lysis buffer (180 μl 20 mM Tris-Cl, 2 mM sodium EDTA, and 1.2% Triton X-100 and 50 μl of lysozyme) was used in step 2, and the incubation time in step 5 was reduced to 10 min. The DNA was quantified using the Qubit fluorometer and sent to the Microbial Genome Sequencing Center (MiGS) at the University of Pittsburgh for library preparation, using the Illumina Nextera kit, and sequencing, using the Illumina NextSeq 550 platform. Sequencing produced 1,565,124 pairs of 150-bp reads. The reads were trimmed using Sickle v1.33 (https://github.com/najoshi/sickle) and assembled using SPAdes v3.13.0 (7) with the “only-assembler” option for k values of 55, 77, 99, and 127. The contigs were annotated using the NCBI Prokaryotic Genome Annotation Pipeline (PGAP) v4.11 (8). The genome coverage was calculated using BBMap v38.47 (https://sourceforge.net/projects/bbmap/). Default parameters were used for all software unless previously stated.

The *S. anginosus* UMB0839 draft genome has a length of 2,104,786 bp and was assembled in 42 contigs. Its GC content is 38.8% with a coverage of 284×. The *N*_50_ score for the genome is 117,003 bp. Annotation with PGAP identified 1,956 protein-coding genes alongside 47 tRNAs and 4 rRNA sequences (2 5S, 1 16S, and 1 23S). Future work will help further our understanding of the role that *S. anginosus* plays in the urogenital microbiota, especially its tendency to cause infection. Moreover, the addition of this strain to the several *S. anginosus* strains already sequenced will help to clarify the genetic characteristics of *S. milleri* bacteria and their identification.

### Data availability.

This whole-genome shotgun project has been deposited in GenBank under the accession no. JAAUWM000000000. The version described in this paper is the first version, JAAUWM010000000. The raw sequencing reads have been deposited in the SRA under the accession no. SRR11441025.
